# Nanopore extended field-effect transistor for selective single-molecule biosensing

**DOI:** 10.1038/s41467-017-00549-w

**Published:** 2017-09-19

**Authors:** Ren Ren, Yanjun Zhang, Binoy Paulose Nadappuram, Bernice Akpinar, David Klenerman, Aleksandar P. Ivanov, Joshua B. Edel, Yuri Korchev

**Affiliations:** 1 Department of Medicine, Imperial College London, London, W12 0NN UK; 2 Department of Chemistry, Imperial College London, London, SW7 2AZ UK; 3 Tianjin Neurological Institute, Tianjin Medical University General Hospital, Heping Qu, 300052 China; 4 Department of Chemistry, University of Cambridge, Cambridge, CB2 1EW UK; 5 National University of Science & Technology MISIS, Moscow, 119049 Russia

## Abstract

There has been a significant drive to deliver nanotechnological solutions to biosensing, yet there remains an unmet need in the development of biosensors that are affordable, integrated, fast, capable of multiplexed detection, and offer high selectivity for trace analyte detection in biological fluids. Herein, some of these challenges are addressed by designing a new class of nanoscale sensors dubbed nanopore extended field-effect transistor (nexFET) that combine the advantages of nanopore single-molecule sensing, field-effect transistors, and recognition chemistry. We report on a polypyrrole functionalized nexFET, with controllable gate voltage that can be used to switch on/off, and slow down single-molecule DNA transport through a nanopore. This strategy enables higher molecular throughput, enhanced signal-to-noise, and even heightened selectivity via functionalization with an embedded receptor. This is shown for selective sensing of an anti-insulin antibody in the presence of its IgG isotype.

## Abstract (web summary)

Efficient detection of single molecules is vital to many biosensing technologies, which require analytical platforms with high selectivity and sensitivity. Ren et al. combine a nanopore sensor and a field-effect transistor, whereby gate voltage mediates DNA and protein transport through the nanopore.

## Introduction

Driven by the need for improved analytical platforms, the development of biosensors has been employed in a wide range of applications from medical diagnostics, drug discovery, environmental monitoring, and rapid pathogen detection to biodefense and environmental surveillance^1^. A wide array of important biological and clinical problems exist that are addressable with biosensors, which could provide positive impact on diagnosing, monitoring, and maintaining health^2^. Nonetheless, most biosensors require extended and potentially complex steps for labeling biomedical analytes with fluorophores, magnetic beads, or active enzymes. Of the many different detection strategies available at present, field-effect transistors (FETs)^3–5^ and nanopores^6^ have emerged among the most attractive single-molecule label-free biosensors. However, both technologies are generally limited by their lack of high selectivity. In addition, FET biosensors are often diffusion limited and rely on passive transport, and, furthermore, the detection sensitivity for large biomolecules is also hampered by the Debye screening length^7–9^. Unlike FETs, nanopore biosensors have the added benefit of allowing active transport, enabling the capture of biomolecules to the lumen of the sensor head once the anlyte is confined within the capture radius^10–12^. However, active and effective nanopore detection of small biomolecules has remained remarkably elusive due to their size and fast transport through the nanopore^13^. Some of these limitations can be addressed by functionalizing the nanopore surface with hydrophobic, and positively or negatively charged residues acting as binding sites^14–17^, which can be used not only to slow down transport but also enable greater selectivity. However, such strategies are often challenging and require careful optimization. It is therefore still a fundamental challenge to develop easy to fabricate and functionalize label-free biosensors that are able to target and measure elusive biological molecules such as nucleic acids, and proteins, with high sensitivity and selectivity while at the same time addressing the limitation described above.

Recently, there has been increasing interest in bringing together both FETs and nanopores to develop ionic-FETs to undertake this challenge^18–21^. The physical principles of ionic FETs are similar to that of the more conventional semiconductor FETs with the exception that the gate medium controls the flow of ions rather than electrons or holes. A potential advantage of using such platforms is that it could enable improved selectivity and controlled molecular transport; however, challenges remain including fabrication, operational stability, and ease of functionalization. A step toward achieving this goal has been in the development of conducting polypyrrole (PPy) FET nanosensors on the tips of multi-barrel nanopipettes^22^.

**Fig. 1:**
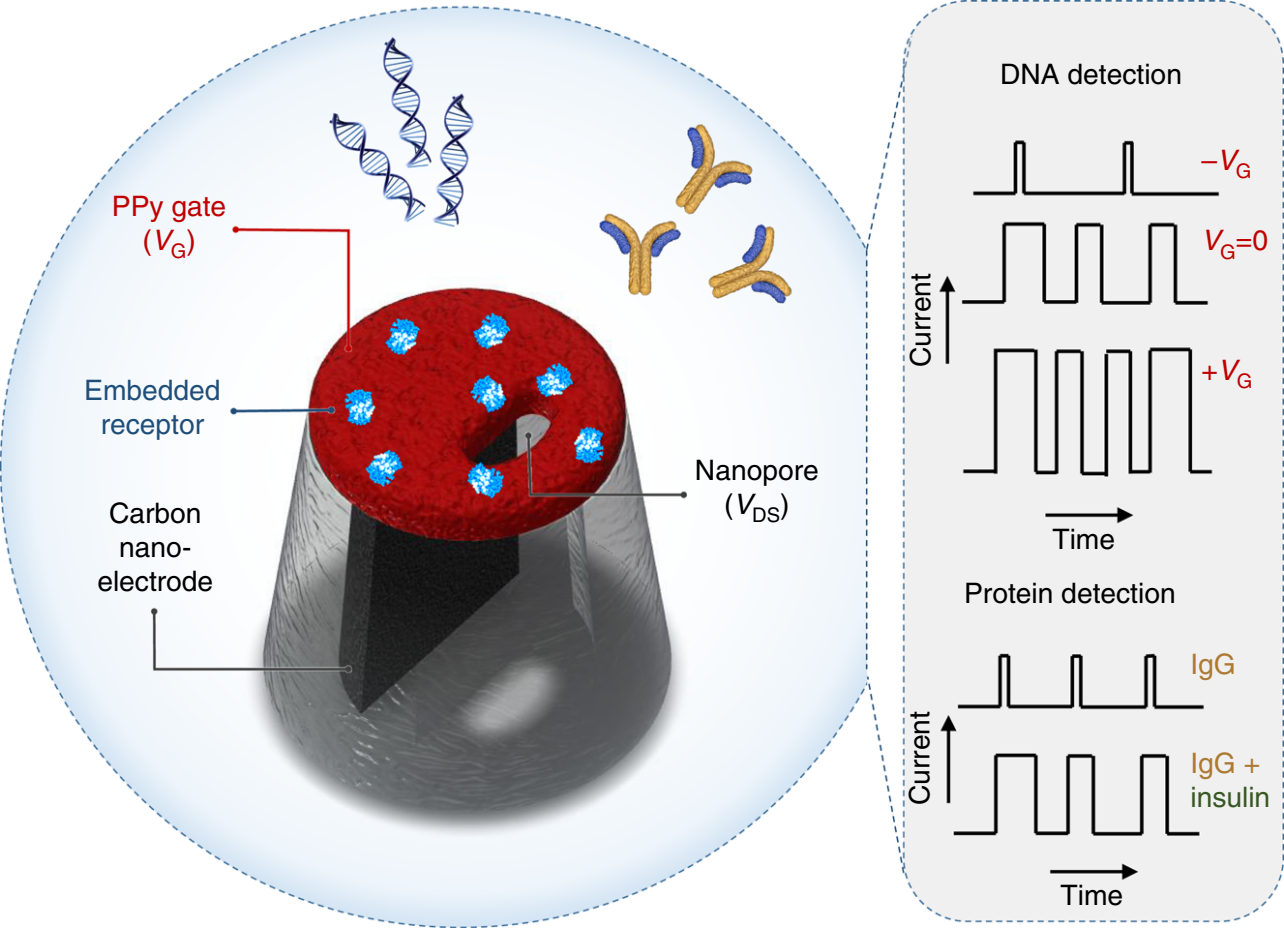
Schematic of the nexFET biosensor. The nexFET platform is a functionalizable ionic nanopore transistor and is based on a dual-barrel quartz nanopipette with one barrel filled with a carbon nanoelectrode that also forms in a localized manner around the pipette tip. The ring-like carbon-electrode surrounding the nanopore is coated with PPy using ionic current feedback controlled electropolymerization, which serves to decrease the opening size of the second barrel. The PPy acts as a gate electrode surrounding the second barrel, a nanopore, that remains open and acts as a drain-source channel. By controlling the gate voltage molecular transport properties and event rates can be efficiently controlled at the single-molecule level. In addition, the PPy gate layer is ideally suited for embedding of artificial receptors that can be used for selective molecular sensing. Both strategies are explored for DNA detection and for protein sensing

Herein, we show that it is possible to combine the advantages from both FET and nanopore platforms, using a novel nanopipette-based PPy ionic-FET, dubbed Nanopore Extended Field-Effect Transistor (nexFET) (Fig. 1). Fabrication of the nexFET is simple and the nanopore dimensions can be tuned in real time to the size of the targeted biomolecule. By controlling the gate voltage we demonstrate that molecular transport can be efficiently controlled at the single-molecule level. In addition, we show that the PPy gate layer is ideally suited for embedding of artificial receptors that can be used for selective molecular sensing.

## Results

### nexFET fabrication and characterization

**Fig. 2:**
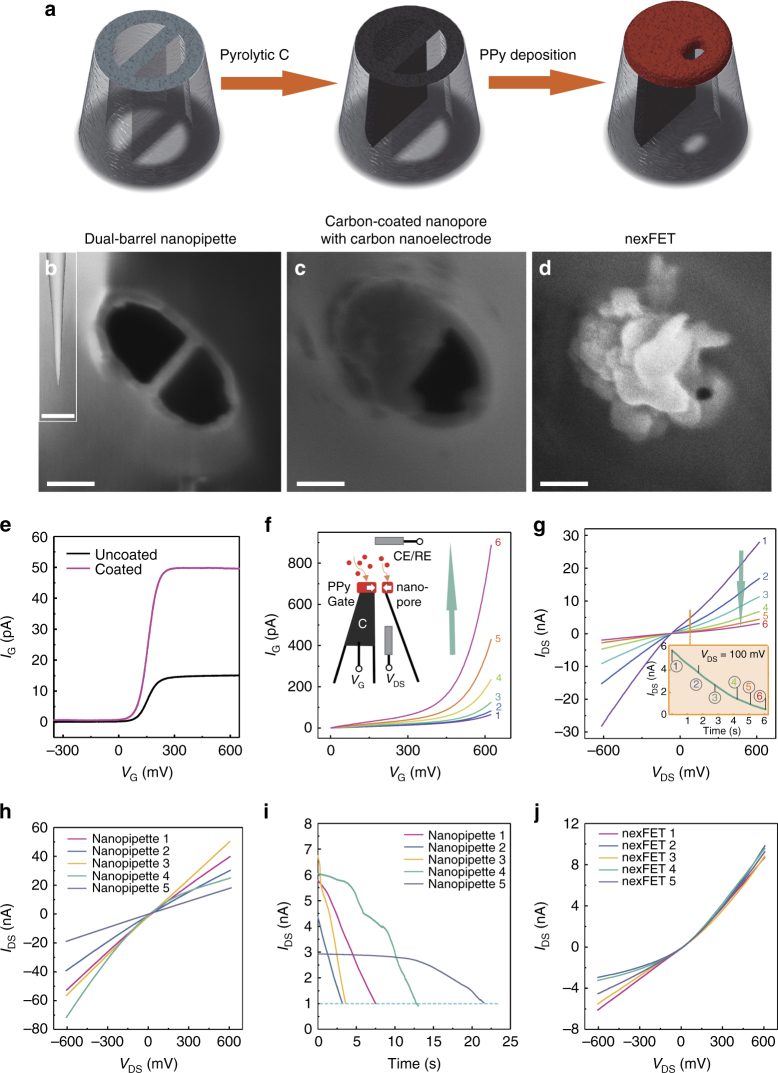
Fabrication and characterization of the nexFET. **a** The nexFET is fabricated in dual-barrel quartz nanopipettes by a pyrolytic carbon deposition in one of the barrels, followed by electrodepositing of PPy at the carbon-coated nanopipette tip. SEM images at each stage of the fabrication process are shown in **b** quartz double barrels after nanopipette pulling (scale bar 100 nm, *inset* 20 μm). **c** One barrel coated with carbon after pyrolytic deposition (*scale bar* 100 nm) and, **d** electropolymerized PPy, acting as a gate electrode, deposited on top of the carbon electrode and surrounding the opening of the nanopore (*scale bar* 100 nm). **e** Linear sweep voltammograms recorded at both carbon surface uncoated and coated tips using 1mM ferrocenemethanol as redox mediator. The three fold increase in limiting current of the surface coated tip confirmed the extension of the carbon electrode around the open barrel for forming the PPy gate. **f** Gate current and gate voltage plots in a representative device as monitored over timer during PPy electropolymerization, showing increasing gate current with each successive PPy deposition cycle (as indicated by the *arrow*). The total deposition time for this particular device was 6 s. The *inset* illustrates the electrode configuration used. **g** Nanopore (drain-source) *I-V*s of the open barrel as monitored during electropolymerization of PPy indicating decrease of the initial nanopore conductance (1) with each successive deposition cycles (2–6) (as indicated by the *arrow*). The *inset* shows the decrease of the initial nanopore current (1) over time with each successive deposition cycle (2–6). **h** *I-V*s for five representative nanopore devices that show very different initial ion transport characteristics. **i** Decrease in nanopore current over time during controllable deposition of PPy until the set-point ionic current (1 nA) is reached for the same devices as in **h**. **j** *I-V*s of the same devices following ionic current feedback deposition confirming reproducibility of the open pore current. Note that there is some variation at negative *V*_DS_ due to geometrical differences in the nanopipettes

The nexFET is fabricated using a double-barrel quartz nanopipette (Supplementary Figs. 1–3) by feedback controlled pyrrole electropolymerization (Fig. 2a–d). Nanopipettes are sub-class of nanopore sensors that have been shown to be easy and rapid to fabricate at low cost^10, 11, 23–26^. Unlike previous reports, we show that a dual-barrel nanopipette can be functionalized and operated as a nanopore and ionic FET in a single platform. In this study, the initial diameter of each nanopipette barrel is ~100 nm, as characterized by scanning electron microscopy (SEM) (Fig. 2b, Supplementary Figs. 1, 2). One barrel was filled with butane gas followed by heating resulting in the pyrolytic decomposition to form a carbon nanoelectrode at the tip and along the surface of the nanopipette (forming a highly localized ring-like carbon-electrode, Fig. 2c–e, Supplementary Fig. 1) for PPy electropolymerization (Fig. 2f). This carbon deposition protocol is similar to that previously published by our group however, in this case we also coated the glass surface in the nanopipette tip with carbon to electrodeposit PPy around the open barrel to form the nanopore. Fig. 2e shows the linear sweep voltammograms (LSVs) recorded at both a carbon surface uncoated and coated tip using 1 mm ferrocenemethanol as the redox mediator. A three-fold increase in limiting current at the surface coated tip, as a result of increased carbon-electrode area, confirmed the presence of carbon surrounding the open barrel necessary for forming the PPy gate around the nanopore.^27^. Importantly the 2nd barrel remains open and acts as the nanopore. The ionic current across the nanopore *I*_DS_ (Fig. 2g) can be used for real-time feedback to control pyrrole deposition, open pore current, and hence nanopore dimension simultaneously as shown in Fig. 2g and Supplementary Fig. 3. Previously, a similar protocol was used by our groups to design metallic nanopores with tunable dimensions^28^. With this method, nanopores with very different initial I-V characteristics (Fig. 2h) can undergo controllable PPy deposition until a set-point ionic current is reached (Fig. 2i). The method yields PPy-coated nanopore devices with very similar ion transport properties (Fig. 2j).

SEM top-view images (Fig. 2, Supplementary Fig. 3) revealed that we have a rough PPy surface morphology yielding nanopores with openings between 10 and 20 nm at the PPy surface. However, function and ion transport through the nanopore depends primarily on the narrowest portion of the pore lumen, which is particularly challenging to image in polymeric-coated pores at these dimensions. A unique method of confirming that the nanopore is within this size regime is to quantify the ion selectivity through the pore. For example, for small sub-10 nm nanopores, it is possible for the diffuse electrical double layers to overlap, which results in high ion selectivity (i.e., counterions easily pass through, whereas coions are repelled)^29, 30^. As shown in Supplementary Fig. 4, this is indeed the regime in which we operate. The ion selectivity was measured as a reversal potential^29^ by using a KCl concentration gradient inside (100 mM) and outside (33.3 mM) of the nanopore. In our devices, after two steps of PPy deposition, the nanopore began to show high ion selectivity, indicating the nanopore dimensions are sub-10 nm’s. Our nexFET nanopores used for the studies in this manuscript were mostly formed after three steps of PPy deposition with a set-point of 1 nA (at 100 mV in 100 mM KCl and 1 mM Tris-EDTA buffer). In principle, this method can be used to tune the open pore current enabling the detection of biomolecule with a large range of sizes.

By modulating the potential applied to the PPy gate, *V*_G_, one alters the distribution of ions in the overlapping electric double layer inside the nanopore. This is apparent when monitoring the ion current rectification (ICR)^31^, where we show that the drain-source current of the nexFET, *I*_DS_, can go from positive to negative rectification simply by controlling *V*_G_. Conventional n-type FETs are electrolyte and pH sensitive and have a stronger field effect at lower bulk salt concentrations^32^. This is consistent with the nexFET where FET gating was observed at KCl concentrations from 1 mM to 1 M (Supplementary Figs. 5, 6). The FET behavior of the nexFET is more pronounced at lower ionic strengths, indicating that the *I*_DS_ may be predominantly governed by changes in the surface charge^19^. Furthermore, the pH dependence (Supplementary Figs. 7, 8) is likely due to the protonation of pyrolitic nitrogen on the PPy in acidic solutions, which results in an increased positive charge on the PPy surface. To demonstrate the versatility of the nexFET, two complimentary applications related to DNA and antibody sensing are shown below, both of which exhibit distinct advantages over more conventional standalone nanopore and FET sensors.

### Single-molecule DNA sensing using a nexFET

**Fig. 3:**
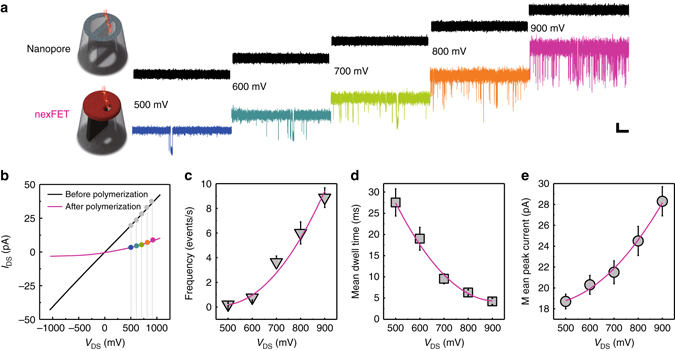
dsDNA translocation behavior through an unmodified and PPy-coated quartz nanopipette. **a** Nanopore detection of 100 pM 3 kbp dsDNA in 100 mM KCl and a 1 mM Tris-EDTA buffer, pH 7 for a quartz nanopipette with a nanopore size of approximately 100 nm (*top*) and nexFET (*bottom*). Each trace is 10 s long and filtered using a low-pass Bessel filter with a cut-off frequency of 10 KHz (*scale bar*: vertical 10 pA, horizontal 1 s). **b** No events are observed in unmodified nanopipette (*black traces* in **a**), due to the large nanopore size as can be seen from the *I-V* curves before and after polymerization. The nexFET with no applied *V*_G_ bias behaves in a very similar manner to a conventional nanopore with **c** increasing event frequency, **d** decreasing mean dwell times, and **e** increasing mean peak currents as the voltage is increased. *Error bars* represent the standard deviation of three independent experimental repeats with different devices

To initially validate the capabilities of the nexFET, a comparison of translocation behavior was performed, before and after PPy polymerization with no *V*_G_ bias applied, using 3 kbp dsDNA at a concentration of 300 pM (Fig. 3). No translocations were observed without PPy. This is due to the starting nanopore size being much larger than what is typically required to obtain observable single-molecule events^6^. After PPy polymerization, the nanopore size was decreased by approximately 90%, which is well within the size regime required to observe single-molecule translocation events. Compared to more conventional solid-state nanopores, longer average dwell times (Fig. 3d) and higher peak current amplitudes (Fig. 3e) were observed. DNA capture was observed to be exponentially dependent on the driving force of the applied voltage, consistent with small nanopores. As in conventional nanopores increasing drain-source voltage resulted in higher peak currents and shorter dwell times.

**Fig. 4:**
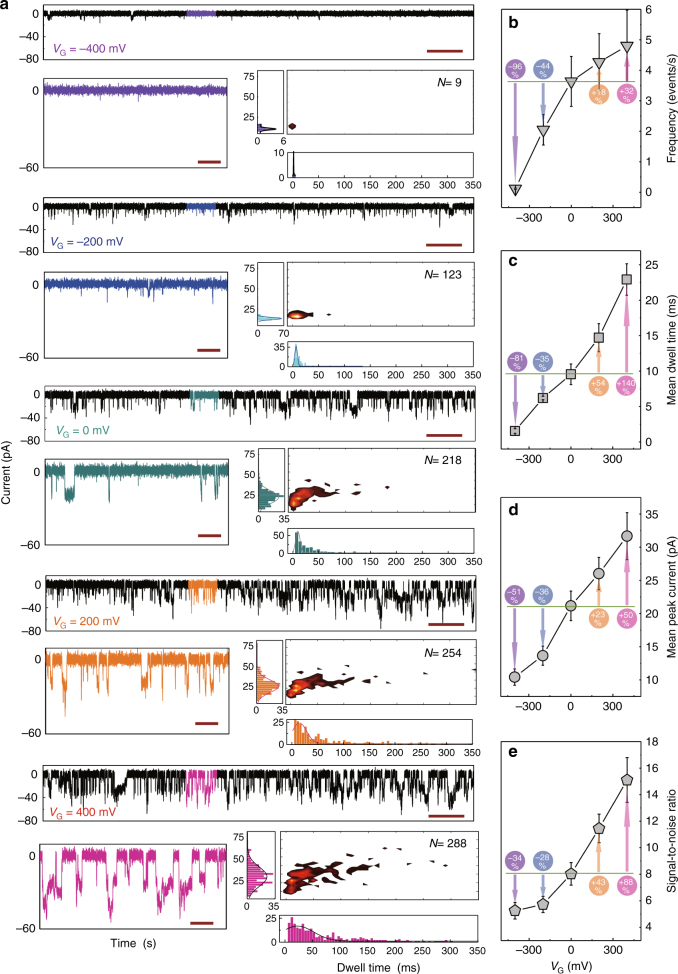
Gate voltage dependence on dsDNA translocation through the nexFET. **a** Representative translocation recordings are shown for 300 pM 3 kbp dsDNA in 100 mM KCl and a 1 mM Tris-EDTA buffer, pH 8 with *V*_DS_ = 700 mV and *V*_G_, from *top* to *bottom*, equal to −400 mV, −200 mV, −0 mV, 200 mV, and 400 mV, respectively. All measurements were conducted at least three times. *I-t* traces are shown for a 60 s duration as well as a 4 s inset. Note that the number of events (*N*) is for the plotted traces. In all cases the traces were filtered using a Bessel filter with a cut-off frequency of 10 KHz (*scale bar*: main traces 5s, partial traces 0.5s). Also included are surface plots along with peak current and dwell time histograms. For a given voltage, **b** the event frequency, **c** mean dwell time, **d** mean peak current, and **e** signal-to-noise ratio were calculated. *Error bars* represent the standard deviation of three independent experimental repeats with different devices

Previously, a number of strategies have been employed to improve translocation throughput, including the use of high salt gradients^12^, application of voltage pulses^10^, or alternatively modification of the internal charge of the nanopore^33^. However, unlike these reports, the nexFET platform allows for control over event frequencies, dwell times, and signal-to-noise ratio simply by modulation of *V*_G_ (Fig. 4). For example, applying a positive bias of *V*_G_ = 400 mV resulted in a 32% increase in event frequency, 140% increase in translocation times, 50% increase in peak current, and 88% increase in the signal-to-noise ratio. When a positive gate voltage was applied to the PPy, the negatively charged DNA chains are attracted and locally concentrated around the nanopore opening. As *V*_G_ increases, the net positive charge on the PPy proportionally increases explaining the observed slowing down of the translocation events (Fig. 4c). The increasing spread in dwell time distributions at higher *V*_G_ can also be rationalized by the increased interaction between PPy and DNA. Furthermore, increasing *V*_G_ leads to a greater propensity for multistep behavior in individual translocation events (Supplementary Fig. 9). The gate voltage was varied from *V*_G_ = −400 mV to *V*_G_ = 400 mV and revealed a linear relationship between increasing *V*_G_ and increasing multistep fraction. While it is possible to attribute this to partially folded DNA or insertions of multiple molecules, it more likely due to the increased electrostatic interaction between the positively charged PPy gate and negatively charged DNA molecules.

Applying a negative bias to *V*_G_ results in a dramatic decrease in event frequency effectively shutting of the nanopore to translocations (Fig. 4b). For example, at *V*_G_ = −400 mV, the mean event frequency and dwell time decreased by 96 and 81%, respectively, relative to *V*_G_ = 0 mV. In summary, we show that it is possible to modulate the event frequency between 0.15 and 4.8 events/s, the mean translocation time between 1.5 and 22.9 ms, and the mean peak current between 10.4 and 31.7 pA. A significant advantage of being able to slow down translocation events and increase the signal-to-noise ratio is in the possibility of detecting short sub-100 base DNA fragments. Such short strands typically translocate too quickly and can only be detected by using more complex nanopore fabrication strategies, such as locally thinning of the membrane^34^ or alternatively by using highly insulating quartz substrates^35^. The nexFET, on the other hand, can be used to detect ssDNA down to 18 bases in length (Supplementary Fig. 10).

### Selective protein sensing using a nexFET

**Fig. 5:**
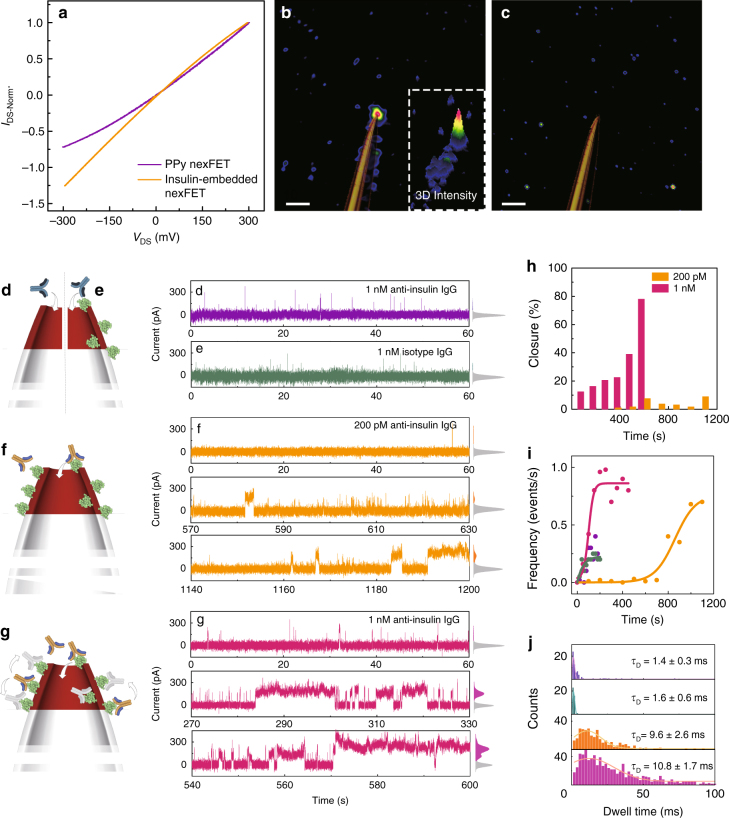
Sensing of anti-insulin IgG antibody with insulin-embedded PPy nexFET. **a** Current-voltage curves for a nexFET with and without embedded insulin. **b** Fluorescence images superimposed with a white light image of the nanopipette confirming selective binding of the anti-insulin IgG antibody to insulin-embedded nexFET by using a fluorescently labeled secondary antibody, **c** and no binding without insulin present (*scale bars*, 10 μm). **d**–**g** Schematics of the proposed translocation mechanism and corresponding current-time traces. Current-time trace for the translocation of **d**, 1 nM anti-insulin IgG antibody through a nexFET without embedded insulin, and **e** 1 nM isotype IgG antibody through an insulin-embedded nexFET. Concentration and time dependence for the translocation of anti-insulin IgG in the presence of an insulin-embedded nexFET is shown in **f** for 200 pM and **g** 1 nM, respectively. For all traces, an all points current histogram is shown on the right-hand side. Histograms of **h** the closure ratio, **i** frequency of translocation, and **j** mean translocation dwell times *τ*_D_ are also shown

Aside from using the PPy as a gate electrode it can also be used as a functional layer by embedding receptors into the surface. For example, the surface of PPy has previously been shown to be functionalized via molecular imprinting and embedding^36^. This is easily achieved by adding the analyte to the PPy precursor prior to electrochemical polymerization. As part of this article we show that it is possible to embed insulin into the PPy, which acts as a receptor for the selective detection of insulin antibodies at the single-molecule level. Figure 5a shows the difference in current voltage characteristics for a nexFET with (100 μM starting concentration) and without insulin embedded in the PPy. Compared with PPy nexFETs, insulin-embedded nexFETs showed negative ICR, indicating successful embedding of the negatively charged insulin protein. To confirm that the insulin activity remains intact, the nexFET was impregnated in a solution consisting of a 1 nM mouse anti-insulin antibody and a fluorescently labeled anti-mouse secondary antibody at the same concentration (Fig. 5b). The control experiments without embedded insulin exhibits no fluorescence after washing (Fig. 5c).

A comparison of current-time traces for the translocation of anti-insulin antibody at *V*_DS_ = −800 mV using both a nexFET and an insulin-embedded nexFET is shown in Fig. 5d–g. Controls are shown for 1 nM IgG anti-insulin antibody without insulin embedded in the nexFET (Fig. 5d) and a non-binding 1 nM IgG isotype antibody with insulin embedded in the nexFET (Fig. 5e). In both cases, it was evident that no binding or interaction took place between the analyte and surface of the nexFET. This is clearly observed in the all points current histogram shown to the right of the traces where by the distribution is dominated by the baseline current. This is not surprising as efficient detection of proteins, without receptor chemistry, is often challenging to detect due to fast translocation times and event rates often being much lower than those predicted from the Smoluchowski rate equation^37^. What is typically detected is the tail end of the distribution, hence much higher protein concentrations (>1 nM) are often required in comparison to DNA.

On the other hand, the insulin-embedded nexFET resulted not only in an increased event frequency but also dwell time, which is due to the affinity between the antigen and antibody^38, 39^. Time-dependent current time traces are shown for 200 pM anti-insulin IgG at *t* = 0, 570, and 1140 s (Fig. 5f) and 1 nM at *t* = 0, 270, 540 s (Fig. 5g). Complete current time traces are shown in Supplementary Figs. 11 and 12, respectively. The rate of events is predominantly governed by diffusion and saturation of the insulin-embedded PPy. Therefore, there is a lag time between the optimal operation of the sensor and the onset at *t* = 0 when the pipette is first inserted in the solution containing analyte. This can be clearly seen in Fig. 5h and i, where the percentage closure (the ratio between the closed and open states of the all points current histogram) and the event frequency are plotted as a function of time. Bound IgG molecules have previously been reported to move and attach to the same or another adjacent antigen within a distance of a few nanometers^40, 41^. When many binding interactions are present at the same time, transient unbinding of a single site is limited and binding of that site is likely to be restored. It is clear that the “closed” state is more dominant for 1 nM than 200 pM and even more so for the two controls where the fraction in the closed state is negligible. The difference in dwell times between the controls and analyte (Fig. 5j) clearly indicate binding in part due to the approximately 5-fold increase in event duration.

## Discussion

Although FET and nanopore sensors have already been shown to be able to perform label-free single-molecule sensing, we demonstrate that it is possible to combine their advantages to improve the sensitivity and selectivity even further for the detection of DNA and proteins. We have shown that nexFET biosensors can successfully detect single-molecule translocation events of DNA and IgG antibodies with longer dwell times, higher capture rates, and improved signal-to-noise ratio when compared to conventional nanopores. Importantly, the interaction between the analyte and the charged PPy surface, either via voltage gating or molecular embedding, plays a large role in the improved performance. For example, in the experiments presented, DNA is thought to weakly bind to the PPy through a combination of non-covalent interactions, in particular the anionic phosphate backbone of DNA is attracted to the cationic PPy and weakly bind together^42, 43^ at the PPy nanopore opening. Some studies also suggested that DNA may deposit and absorb to the PPy surface^42, 44^; however, permanent attachment of DNA on PPy was not observed as the applied electric field in *V*_DS_ can be used to control the desorption of DNA. We also show that with the nexFET it is possible to selectively detect IgG antibodies in the presence of embedded insulin in the PPy.

A significant advantage of the nexFET is in the real-time tuning of the nanopore ion transport characteristics, which introduces the possibility of adapting pore dimensions to that of the analyte by performing feedback controlled electropolymerization. This has extensive implications in optimization of throughput and at the same time improvement of the signal-to-noise ratio. The ability to apply FET gating control to further tune nanopore surface charge and ion transport in combination with molecular imprinting render nexFETs as a very versatile biosensor platform that can be adapted for analyte-specific screening.

## Methods

### Fabrication of the dual-barrel nanopipette

Double-barrel quartz theta capillaries (o.d., 1.2 mm, i.d., 0.9 mm, Intracell) were plasma cleaned (Harrick Plasma), and pulled with a laser-based P-2000 pipette puller (Sutter Instruments) using a single-line program (heat 700, filament 3, velocity 45, delay 130, and pull 93) to produce sharp nanopipettes with individual barrel diameters of approximately 100 nm at the tip as characterized by SEM and TEM imaging. It should be noted that the above pulling parameters are instrument specific and variations will exist from puller to puller.

### Fabrication of nanopipette with one carbon nanoelectrode

A previously published protocol developed by our groups was used^27^. Briefly, one barrel of the dual-barrel nanopipette was filled with propane/butane and heated under inert atmosphere (Argon) with a butane flame to decompose the carbon gas and yield pyrolytic carbon inside the nanopipette. In order to ensure the tip is carbon-coated it is important to heat the barrel of the pipette for a minimum of 15 s; this results in an electrode area slightly larger than the area of a single barrel. The entire fabrication process takes approximately 1 min per electrode. The quality of carbon electrode was assessed by using cyclic voltammetry (CV) to characterize the redox behavior of 1 mM ferrocenemethanol in 100 mM KCl (Sigma-Aldrich).

### PPy deposition

Pyrrole (Sigma-Aldrich) was used as received and stored under argon atmosphere. PPy was electrodeposited onto the carbon electrode by applying a 600 mV potential using a deposition solution consisting of 500 mM pyrrole, 200 mM lithium perchlorate (Sigma-Aldrich), and 100 mM perchloric acid (Sigma-Aldrich) in water. The electrochemical behavior of the PPy nanopore formed in the double-barrel nanopipette was characterized by CV. The deposition was monitored via real-time current feedback through the open barrel, *V*_DS_ = 100 mV. The ionic current is linked to the nanopore dimensions, hence monitoring the current in real time is useful for ensuring reproducibility from pipette to pipette. Prior to usage, all nexFET’s were cycled between −300 and 300 mV in 100 mM HCl until stable currents were obtained. All experiments were performed in Tris-EDTA pH-buffered solutions.

### Fabrication of insulin-embedded nexFETs

Insulin-embedded nexFET were prepared by electropolymerization at 600 mV using a 500 mM pyrrole solution containing 100 μM human insulin (Sigma-Aldrich), and 100 mM KCl buffered at pH 6 using Tris-EDTA. Before usage, the nexFET was gently washed several times with Tris-EDTA buffer until the drain-source currents were stable.

### Ionic currents measurement

The nexFET performance and time-dependent ionic current recordings were measured using a Multiclamp700B amplifier and DigiData 1322A digitizer (Molecular Devices). Ag/AgCl electrodes were inserted into the bath (ground electrode) and the nanopipette ionic channel (working electrode) to monitor the drain-source currents. In addition, a silver wire was embedded into the carbon electrode, which was used for both PPy electropolymerization, and application of the gate voltage. The data were recorded and analyzed using pClamp software (Molecular Devices).

### DNA and protein translocation experiment

DNA translocation experiments were performed using 300 pM, 3 kbp dsDNA dissolved in a 100 mM KCl solution containing 1 mM Tris-EDTA (pH = 8). Anti-insulin IgG antibody translocation experiments were carried out using 1 nM anti-insulin IgG antibody (BSA and azide free, ab46707, Abcam) dissolved in a 100 mM KCl solution containing 1 mM Tris-EDTA (pH = 7.0). Depending on the experiment, either DNA or protein was loaded into the bath, while the open barrel of the nexFET was filled with the buffer of the same composition. The current time traces were filtered with a 10 kHz low-pass filter and translocation events were analyzed using Clampfit and a custom-written MATLAB code developed in-house.

### Data availability

The data that support the findings of this study are available from the corresponding author upon request.

## Electronic supplementary material

pdf si with si guide on the first page
