# Supplementary material for: Nanopore extended field-effect transistor for selective single-molecule biosensing
Source: Nat Commun. 2017 Sep 19;8:586. doi: 10.1038/s41467-017-00549-w (PMC5605549; doi:10.1038/s41467-017-00549-w)
Supplement: Supplementary file 1 — pdf si with si guide on the first page [file 41467_2017_549_MOESM1_ESM.pdf]

Description of Supplementary Files

File name: Supplementary Information
Description: Supplementary figures

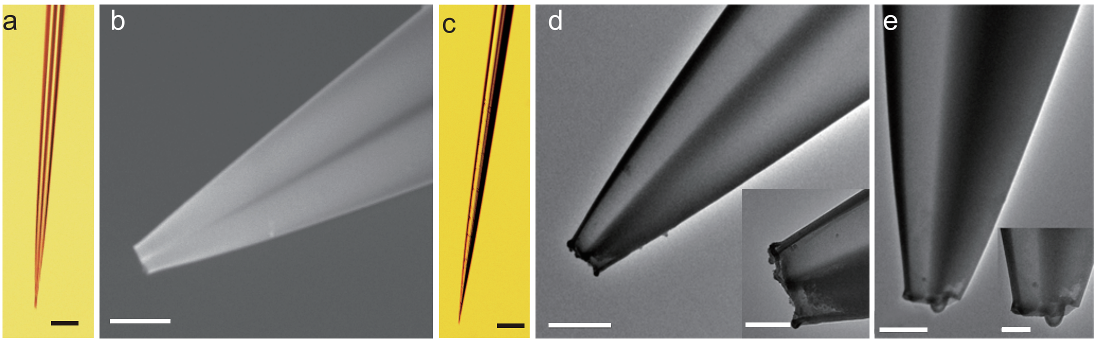

Supplementary Figure 1: Nanopipette imaging.

Optical microscopy side-view image (scale bar: 100 µm) (**a**) and SEM image (**b**) of a typical dual-barrel nanopipette prior to pyrolytic carbon deposition. Optical microscopy side-view image (scale bar: 100 µm) (**c**) and TEM images (**d, e**) of a nanopipette with one carbon filled barrel (darker barrel). The bare double-barrel nanopipette terminate with a sharper edge, while the deposited carbon tends to form uneven ring-like (or crown-like) carbon deposits localized at the tip. All TEM images have a scale bar of 500 nm and insets 200 nm.

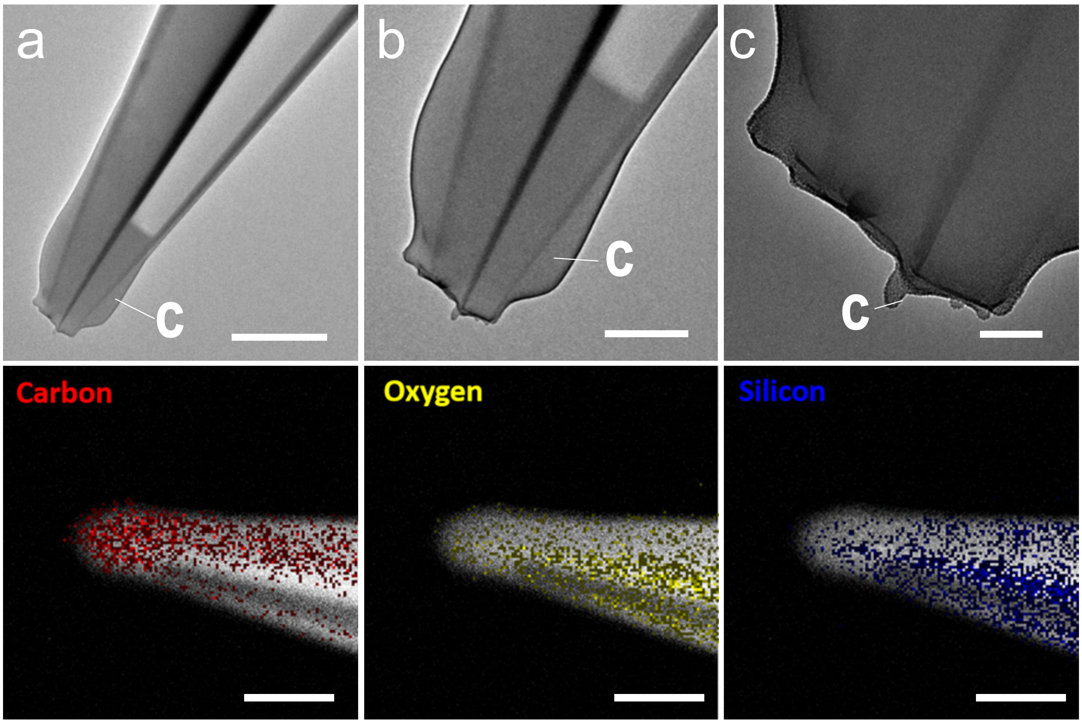

Supplementary Figure 2: **Elemental analysis of a carbon-deposited nanopipette.**

(**a-c**) TEM images and elemental analysis of a carbon-deposited nanopipette (scale bar: a: 1 µm, b: 200 nm, c: 100 nm). For the purposes of elemental analysis carbon was over- deposited (higher butane flow-rate and lower argon flow-rate were used during pyrolytic deposition), which confirmed that carbon deposition occurs on the tip of the nanopipette. Here ring-like (or crown-like) carbon structures are clearly observed on the tip of the nanopipette. Element mapping analyses show the existing of (**d**) carbon, (**e**) oxygen, and (**f**) silicon distribution at the tip of the nanopipette ((scale bar: 1 µm).

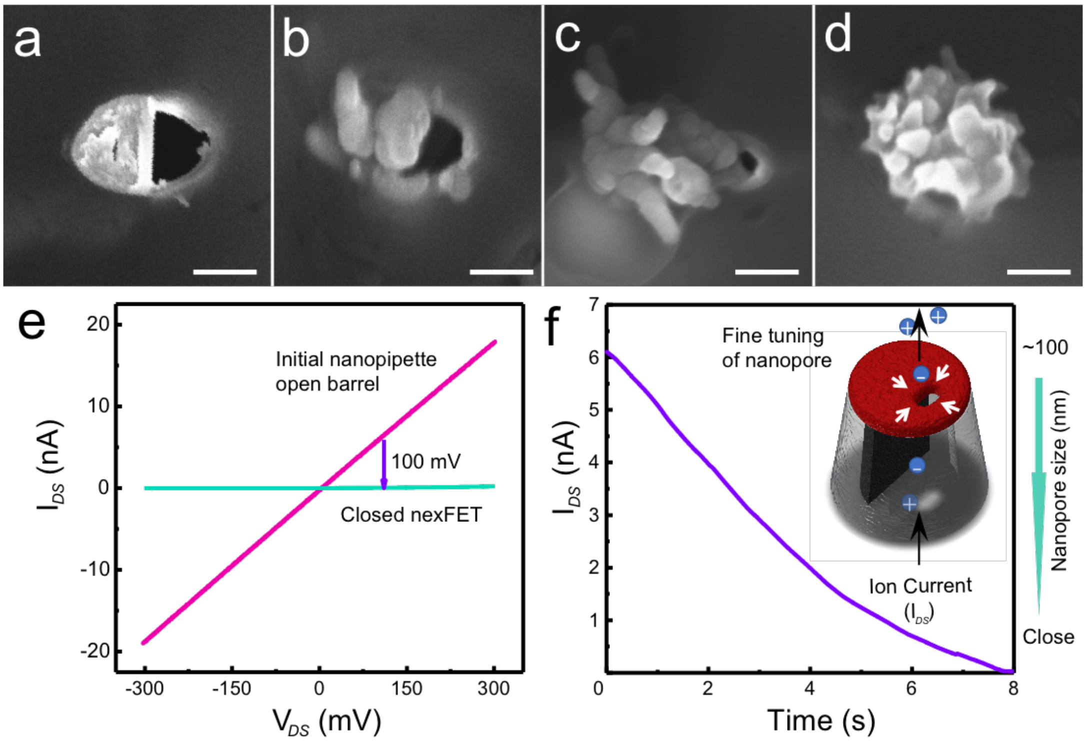

Supplementary Figure 3: **Nanopipette characterization at different stages of nexFET fabrication**. SEM images of (**a**) carbon-filled nanopipettes with a 100 nm open barrel, (**b**) nexFET with ~80 nm nanopore after PPy deposition, **(c)** nexFET with ~ 10 nm nanopore after PPy deposition, and **(d)** a fully blocked nexFET after longer polypyrrole deposition. (**e**) representative I-V plots showing the change from an open barrel nanopipette (red color) to a closed nexFET after 8 seconds of PPy deposition (green color). (**f**) Nanopore current I_{DS} (at 100 mV applied voltage) was measured over time to monitor decreasing nanopore conductance during PPy deposition until the nanopore was fully closed within 8 seconds. (scale bars: 100 nm).

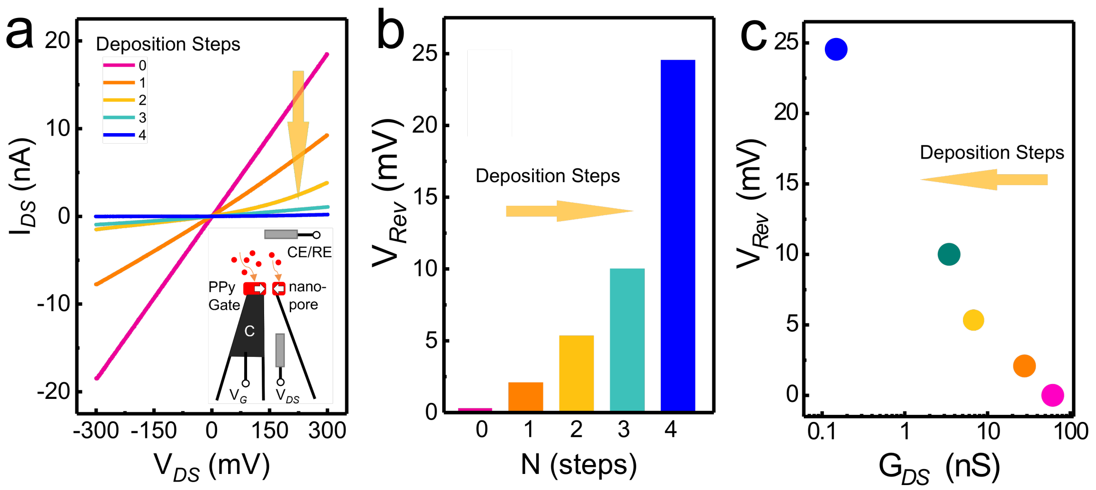

Supplementary Figure 4: nexFET ion selectivity characterization.

(**a**) I-V plots, and **(b-c)** ion-selectivity of nexFET nanopore at different steps of PPy deposition on the tip of the nanopipette. The ion selectivity is measured as a reversal potential with a 3 times KCl concentration gradient (inside/outside ratio = 100 mM/ 33.3 mM) across the nexFET. The deposition steps are colour coded identically in all plots. Step 0 is the initial drain-source I-V of the open-barrel of the nanopipette before PPy deposition. Following deposition of PPy on the tip, the ion current rectification ratio and ion selectivity increases, while nanopore conductance decreases.

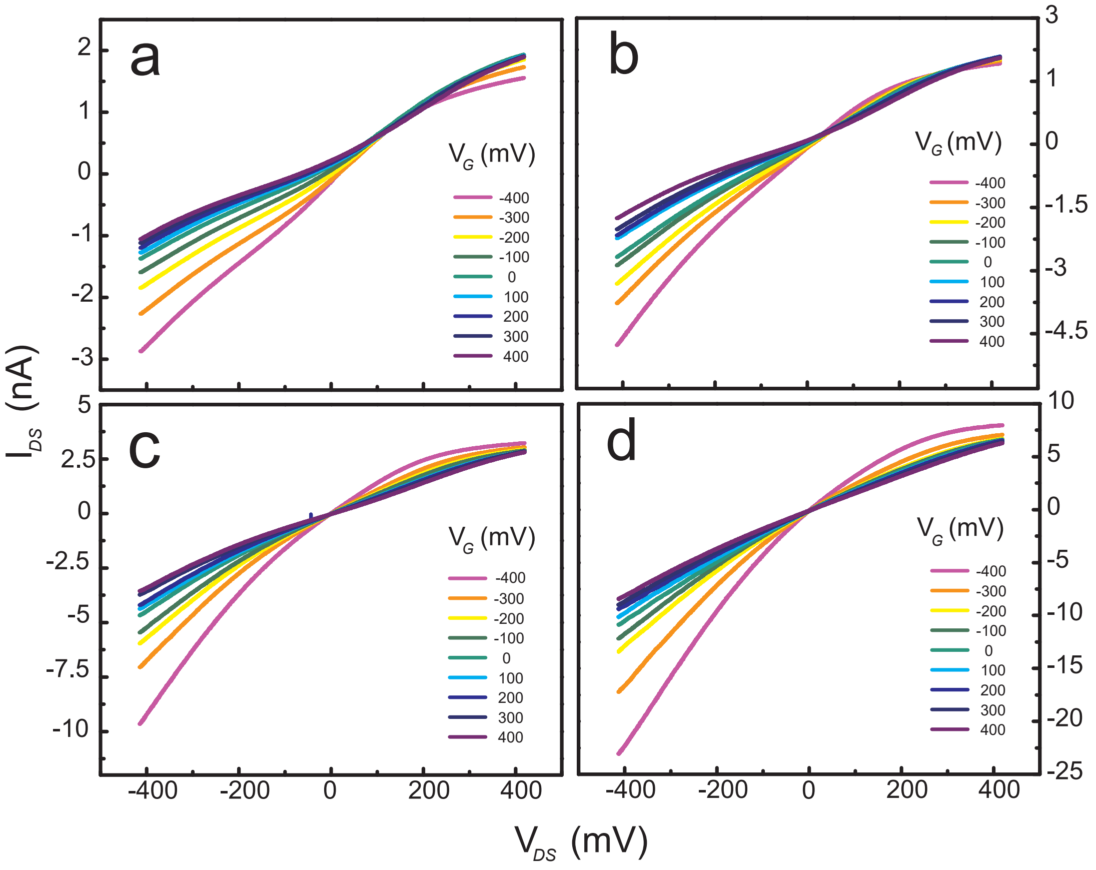

Supplementary Figure 5: Ionic transport (nanopore) dependence on applied gate voltage in the nexFET.

I_{DS}-V_{DS} characteristics were measured at KCl concentration of (**a**) 1 mM, (**b**) 10 mM, (**c**) 100 mM, and (**d**) 1 M, respectively.

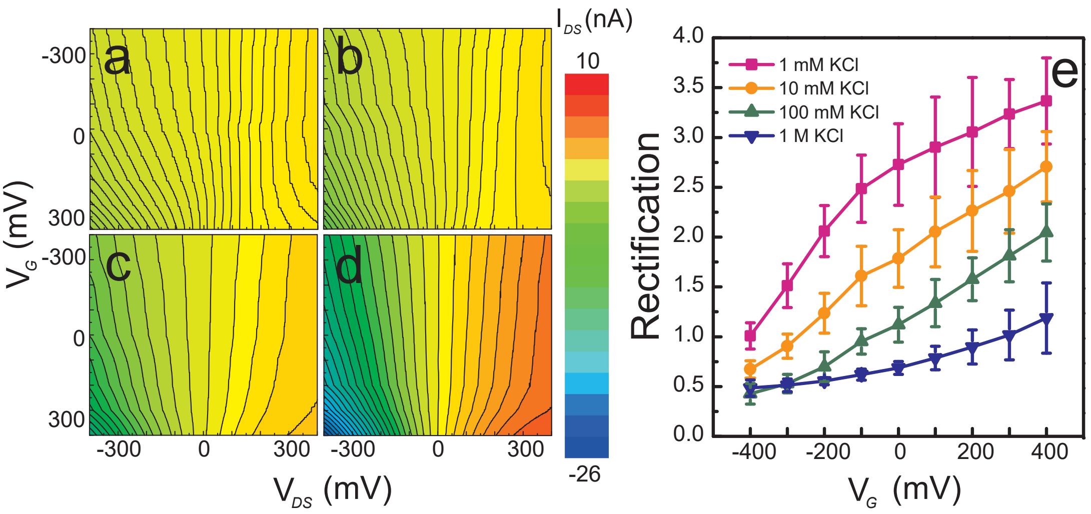

Supplementary Figure 6: **Ionic transport (nanopore) dependence on applied gate voltage in the nexFET (contour plots).**

(**a-d**) Contour plots (V_G vs V_{DS}) for the I_{DS}-V_{DS} plots shown in Fig.S5. (**e**) Ion current rectification dependence on applied gate voltage (V_G) at different concentrations of KCl ranging from 1 mM to 1 M. Error bars represents the standard deviation of 3 times independent experiment.

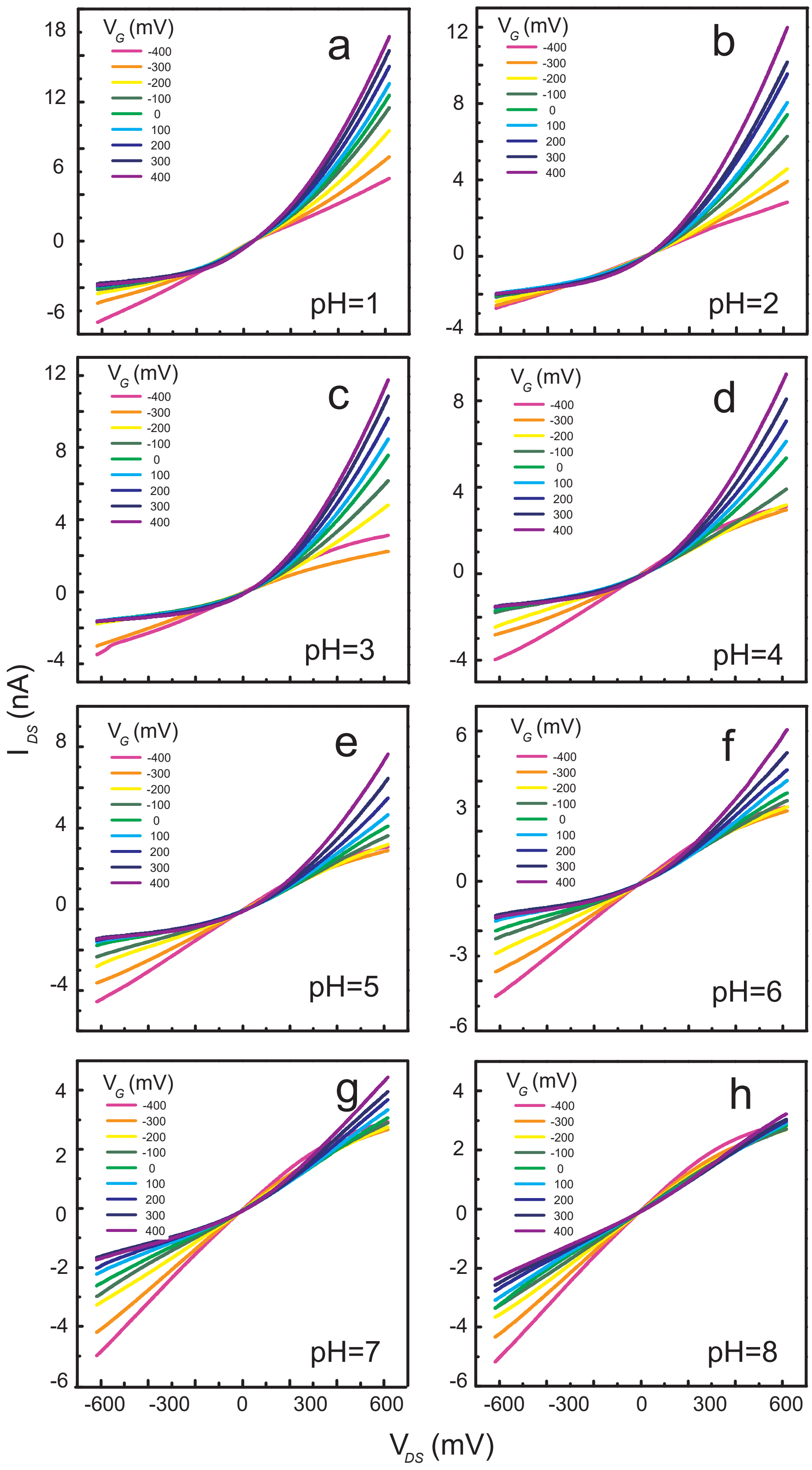

Supplementary Figure 7: Ionic transport (nanopore) dependence on pH.

(**a-h**) I_{DS}-V_{DS} curves demonstrating the pH dependence of the nexFET at different gate voltages. The representative I_{DS}-V_{DS} characteristics were measured from pH 1 to pH 8 (**a-h**) at gate voltages (V_G) ranging from -400 to +400 mV.

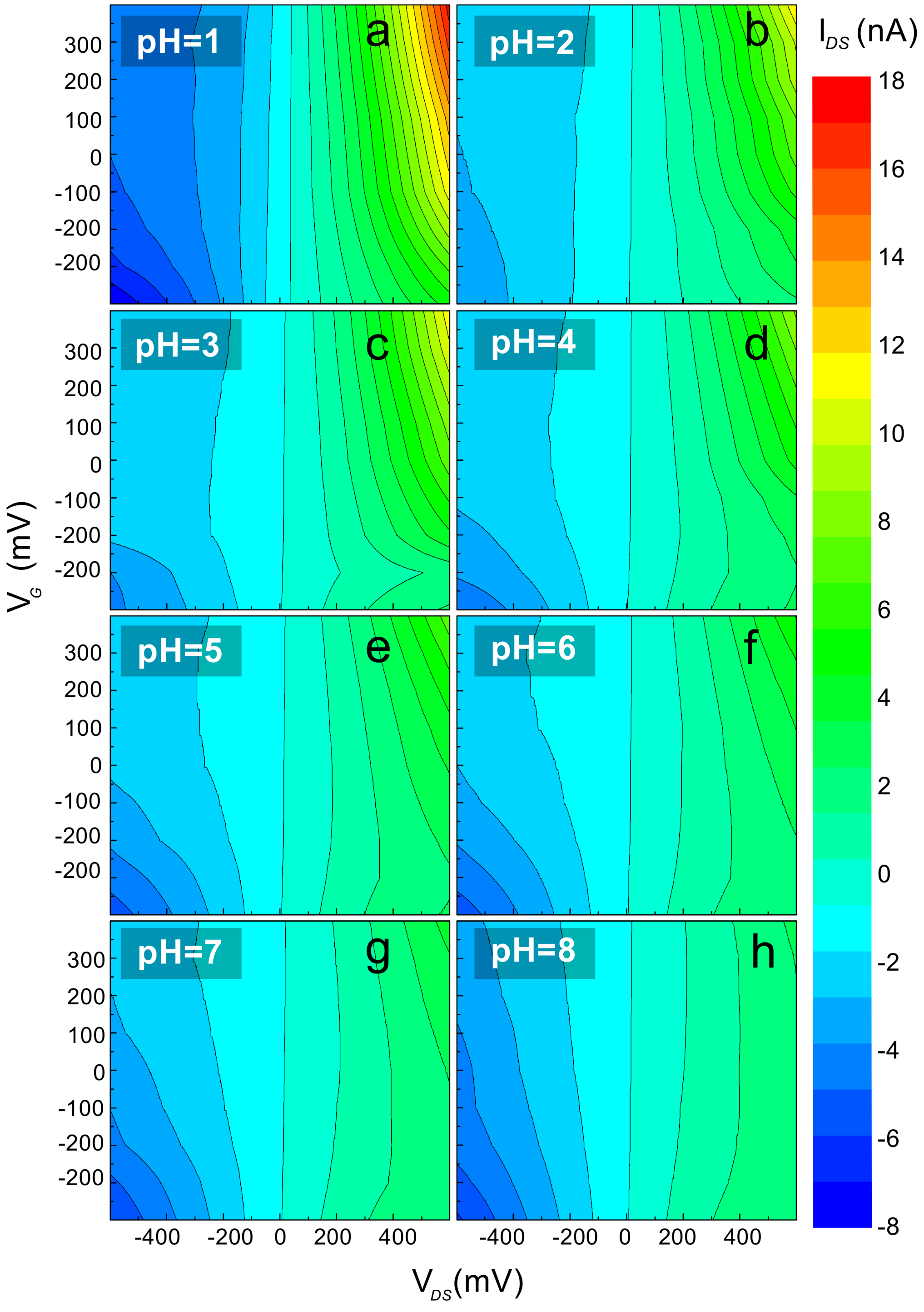

Supplementary Figure 8: Ionic transport (nanopore) dependence on pH (contour plots)
(**a-h**) Contour plots (V_G vs V_{DS}) for the I_{DS}-V_{DS} plots shown in Fig. S7.

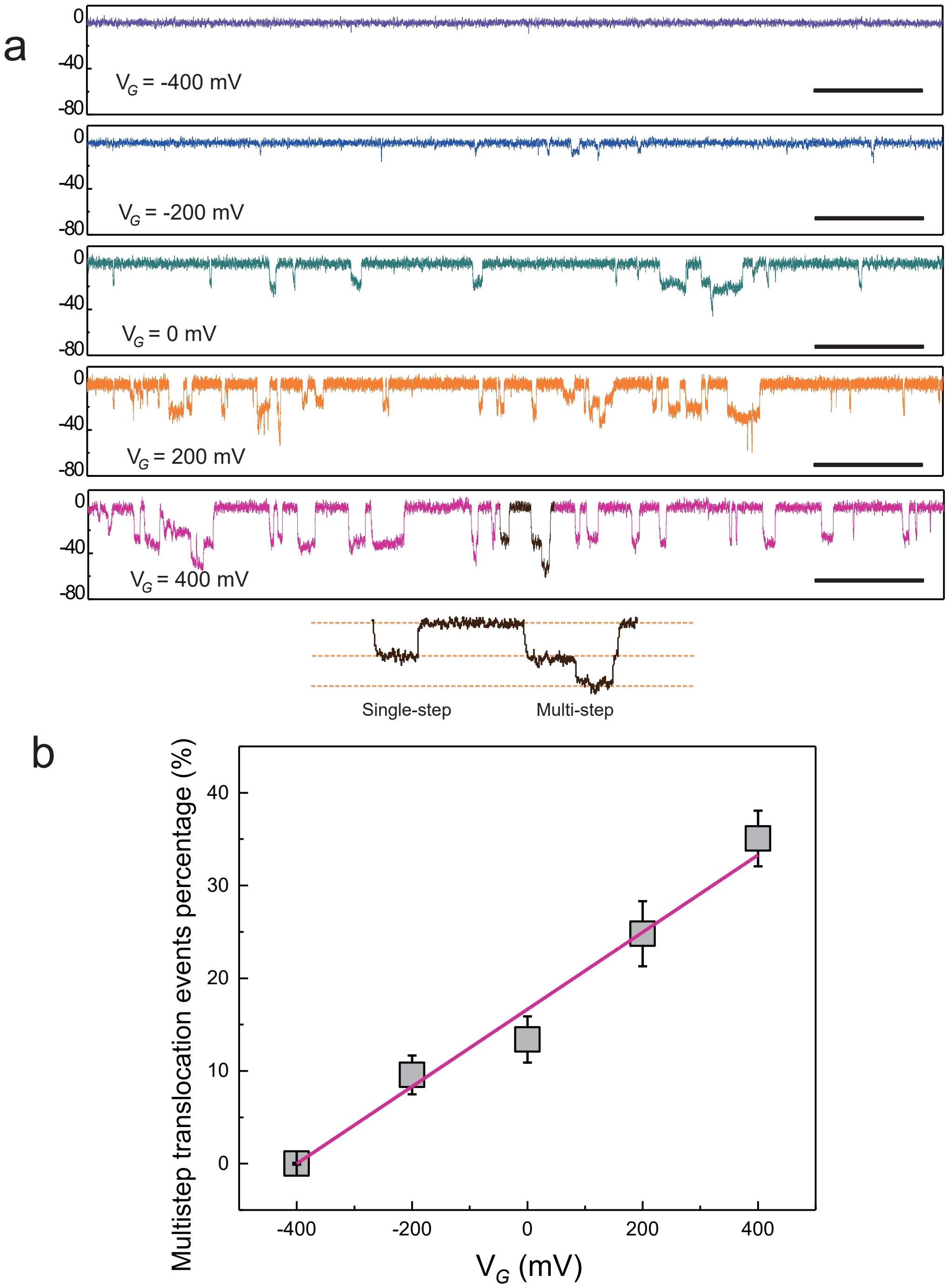

Supplementary Figure 9: Ratio of multi-step events in DNA individual translocation events at different applied gate voltages.

(**a**) Representative I-t trace showing individual single and multistep events for 300 pM 3 kbp dsDNA in 100 mM KCl and a 1 mM Tris-EDTA buffer, pH 8 with V_{DS} = 700 mV (scale bar: 0.5 s). (**b**) Increasing V_G leads to an increased fraction of multisteps. The gate voltage was varied from V_G =-400 mV to V_G = 400 mV and revealed a linear relationship between increasing V_G and increasing multistep fraction. This was attributed to an increased electrostatic interaction between the positively charged PPy gate and negatively charged DNA molecules. Error bars represents the standard deviation.

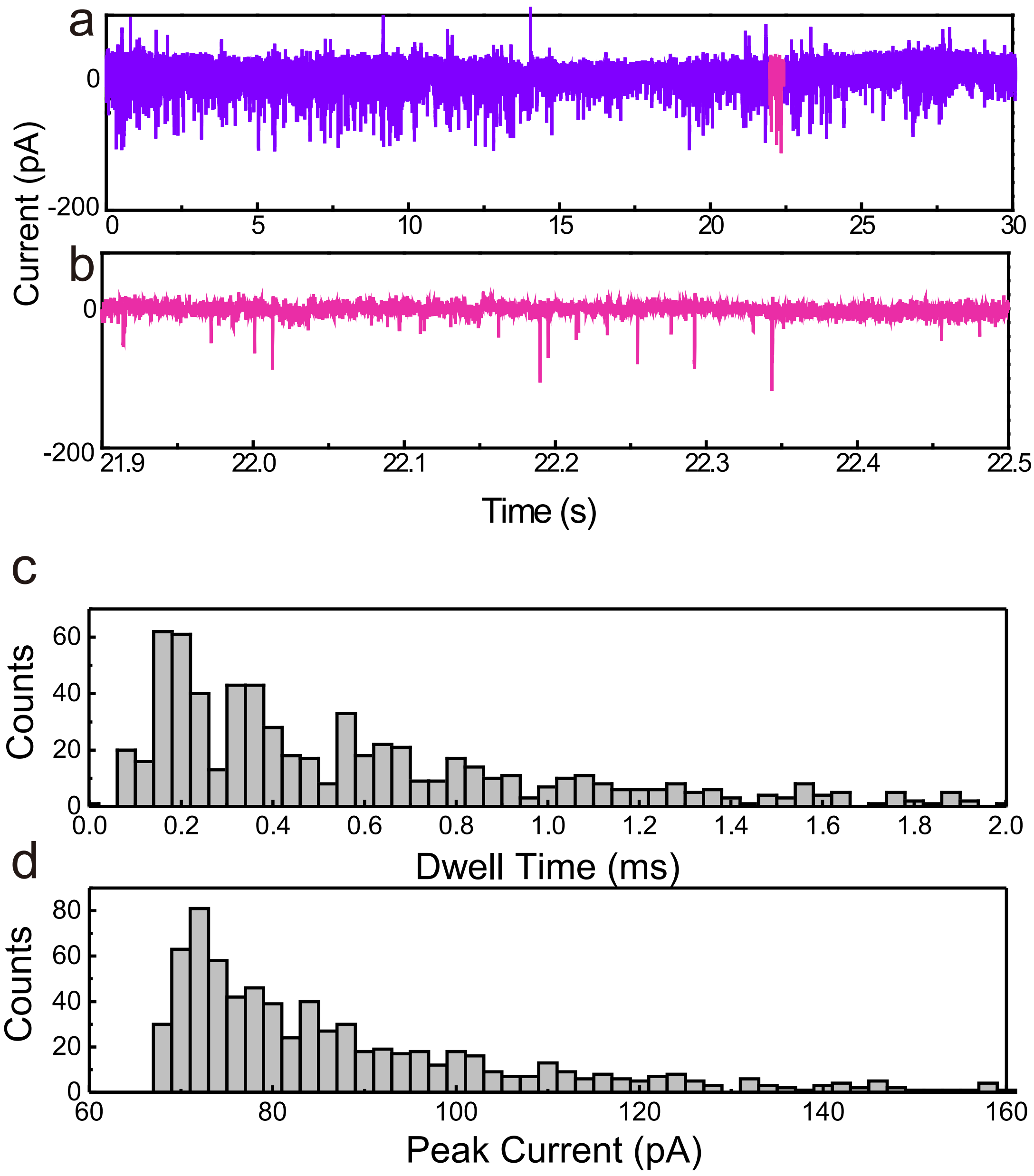

Supplementary Figure 10 | Detection of single molecule translocation events for single strand DNA (ssDNA). **(a)** I-t traces of 1 nM ssDNA (18 bases) detection in 100 mM KCl and 1 mM Tris-EDTA (pH=8) buffer under applied drain-source voltage of 700 mV. (b**)** Zoomed in trace showing individual translocation events. Dwell time (mean: 0.22 ms) and current amplitude (mean: 73.85±8.56 pA) histograms are shown in **c)** and (**d),** respectively.

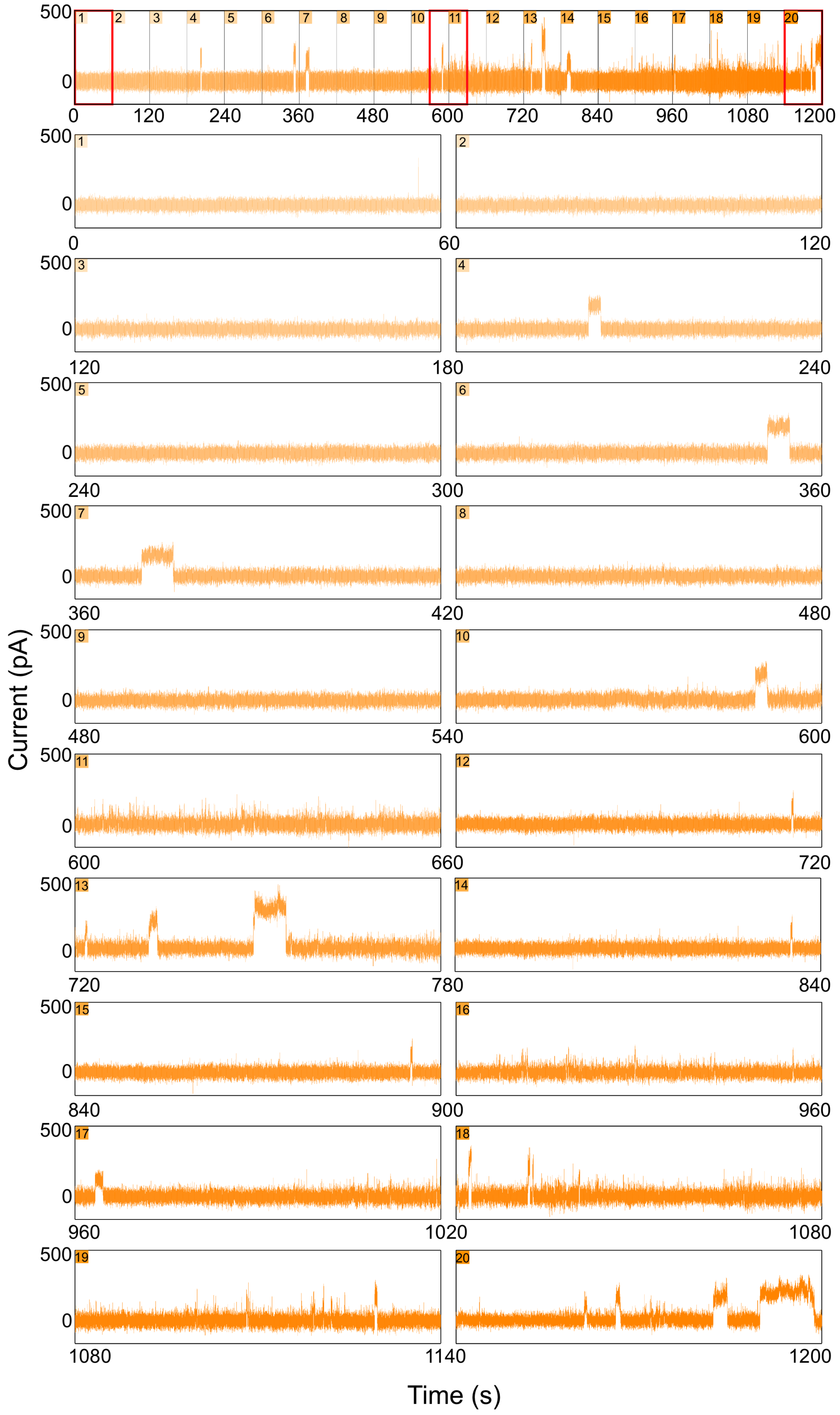

Supplementary Figure 11: 200 pM anti-Insulin IgG antibody through an insulin embedded nexFET. 1200 s full I-t trace is shown for the detection of 200 pM anti-Insulin IgG antibody with an insulin embedded nexFET in 100 mM KCl, 1mM TE, pH=7 solution under -800 mV drain-source voltage. The top panel shows the full trace while the remaining 20 panels show zoomed 60 s successive traces. The initial (0-60 s), the middle (570-630 s), and the end (1140-1200 s) segments of the trace are shown in the main manuscript Fig. 5 f. The histograms of closure ratio (Fig. 5 h), frequency of translocation (Fig. 5 i), and translocation dwell time (Fig. 5 j) for 200 pM antibody are calculated from this full trace.

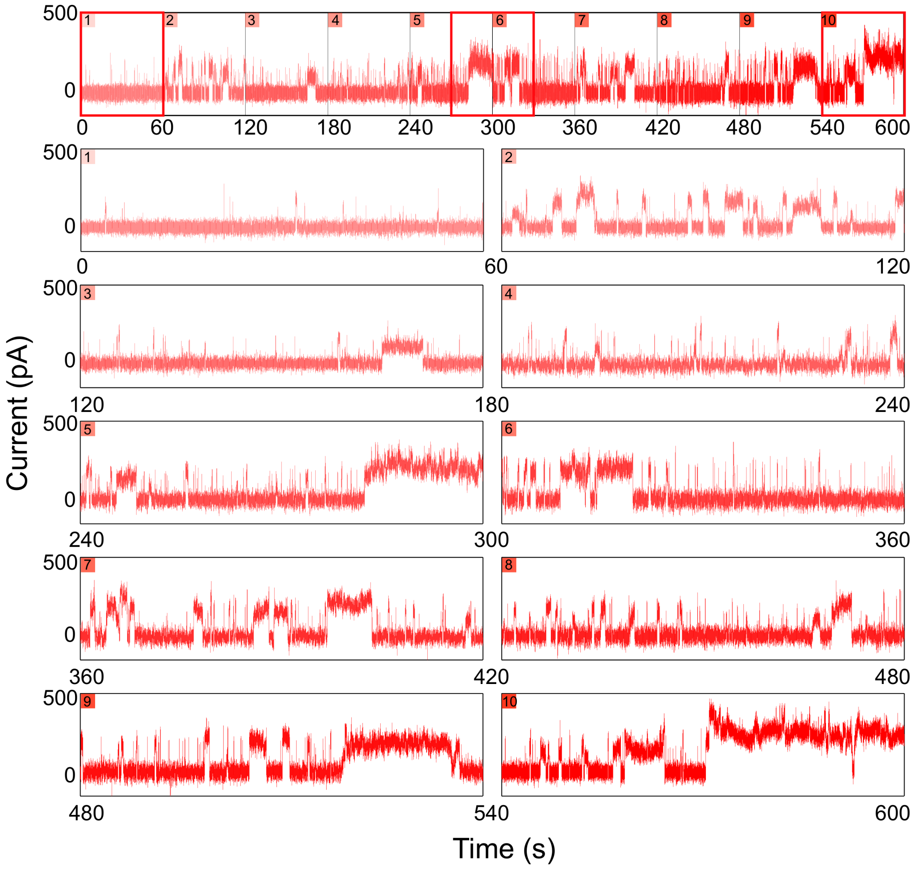

Supplementary Figure 12 | 1 nM anti-Insulin IgG antibody through an insulin embedded nexFET. 600 s full I-t trace is shown for the detection of 1 nM anti-Insulin IgG antibody with an insulin embedded nexFET in 100 mM KCl, 1mM TE, pH=7 solution under -800 mV drain-source voltage. The top panel shows the full trace while the remaining 10 panels show zoomed 60 s successive traces. The initial (0-60 s), the middle (270-330 s), and the end (540-600 s) segments of the trace are shown in main manuscript Fig. 5 f. The histograms of closure ratio (Fig. 5 h), translocation frequency of translocation (Fig. 5 i), and translocation dwell time (Fig. 5 j) for 1 nM antibody are calculated from this full trace.
